# Two-Thirds of Smear-Positive Tuberculosis Cases in the Community Were Undiagnosed in Northwest Ethiopia: Population Based Cross-Sectional Study

**DOI:** 10.1371/journal.pone.0028258

**Published:** 2011-12-02

**Authors:** Takele Tadesse, Meaza Demissie, Yemane Berhane, Yigzaw Kebede, Markos Abebe

**Affiliations:** 1 School of Public Health, University of Gondar, Gondar, Ethiopia; 2 Addis Continental Institute of Public Health, Addis Ababa, Ethiopia; 3 Armauer Hansen Research Institute, Addis Ababa, Ethiopia; McGill University, Canada

## Abstract

**Background:**

Tuberculosis (TB) case detection rate remains low in Ethiopia. One of the underlying reasons is the emphasis on passive case finding strategy which may seriously underestimate the burden of the disease. Estimating the prevalence of smear-positive pulmonary TB through active case finding at population level can help assessing the degree to which passive case detection is successful.

**Methods and findings:**

This is population based cross-sectional study. The study population was all individuals aged ≥14 years. Interviews using a uniform questionnaire were done initially to identify individuals with chronic cough (≥15 days) and the two sputum (spot and morning) samples were gathered for standard smear microscopy. A total of 23,590 individuals aged ≥14 years were interviewed and 984 had a chronic cough for ≥15 days. Of 831 individuals who provided two sputum samples for acid fast bacilli (AFB), 41 had positive smears. A total of 22 smear-positive TB cases detected through passive case finding were on anti-TB treatment. The prevalence of new smear-positive TB was 174 per 100,000 in persons aged ≥14 years (95% CI: 121–227).The ratio of active to passive case finding was 2∶1. Higher rates of smear-positivity were observed among females [AOR: 3.28, 95% CI (1.54–6.77)], and in the age group ≥45 years [AOR: 2.26, 95% CI (1.12–4.59).

**Conclusions:**

The study revealed that about two-thirds of patients with active TB remain undiagnosed and thus untreated. This may indicate the need for strengthening case detection at the community level. Furthermore, the high burden of TB among females and in the age group ≥45 years warrants appropriate measures to control the disease.

## Introduction

Tuberculosis (TB) remains one of the major causes of morbidity and mortality worldwide, while TB control strategy Directly Observed Treatment Short-course (DOTS) is one of the most cost-effective interventions [1]. More than 81% of TB cases and deaths comes from developing countries; the TB situation in those countries is aggravated by high prevalence of human immunodeficiency virus, drug resistance, social inequalities, poor TB control efforts and inadequate health care spending [2], [3]. However, very few population based TB prevalence studies were carried out in these countries [4]–[9].

Ethiopia ranked seventh among world's 22 high burden countries, with smear-positive TB case notification rate of 57 per 100,000 population [11] and 54 deaths per 100,000 population [1].The National Tuberculosis and Leprosy Control Program began to implement DOTS in 1994 [10]. In 2010, DOTS coverage is reportedly reached 100% and TB treatment is integrated into general health services. However, TB case detection rate remains very low in Ethiopia (36%) [11]. One of the underlying reasons is the emphasis only on passive case finding strategy, where TB patients self-report to health care facilities which are not equipped with the necessary diagnostic facilities and qualified health professionals. This strategy has the potential to seriously underestimate the burden of the disease and many TB cases remain undetected and thus untreated creating more chances for TB spread in the community [12]–[17]. Some interpreted the low detection rate as a sign of declining prevalence of TB rather than failure to detect cases. Population based studies can help to resolve such controversies. Therefore, this study aimed at estimating the prevalence of smear-positive TB through active case finding in Northwest Ethiopia.

## Methods

### Study setting and population

**Figure 1:**
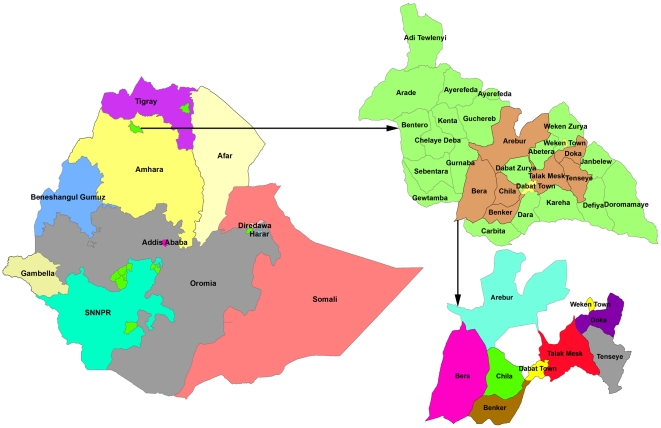
Map of the study area at Dabat district in Northwest Ethiopia. Area shaded with different colors-Dabat HDSS.

The study was conducted at Dabat Health and Demographic Surveillance System (HDSS) hosted by the University of Gondar located at Dabat district in Northwest Ethiopia (see map, Figure 1). Dabat district has an estimated population of 145,458 living in 27 rural and 3 urban Kebeles (the smallest administrative unit in Ethiopia). The local communities are largely depend on subsistence agriculture economy. The district has two health centers, three health stations, and twenty-nine health posts providing health services for the community. Only the two health centers provide DOTS for TB cases [18]. The Dabat HDSS covers ten randomly selected Kebeles with a total population of about 46,165, of which 23,590 (51%) were adults aged ≥14 years. Information on vital events like birth, death, and migration are collected quarterly [19]. Active TB surveillance was introduced at HDSS in collaboration with the district health office in October 2010.

### Study design

Population based cross-sectional study was conducted to estimate the prevalence of smear-positive TB at Dabat district from October to December 2010.

#### Data collection

Eighteen data collectors and three supervisors from Dabat HDSS were trained on pulmonary TB screening. They interviewed all individuals aged ≥14 years using pre-tested and structured symptom screening questionnaire to identify pulmonary TB suspected cases. Persistent cough for ≥15 days with or without other related symptoms was taken as a cardinal symptom for pulmonary TB. They also interviewed each individual whether he or she was on anti-TB treatment. Socio-demographic, geographical, and history of current and previous illnesses information were collected from each individual. After obtaining written informed consent, data collectors gathered two sputum samples (spot and morning) from every subject reported chronic cough with sputum using sputum cup labeled with unique individual identification number. The sputum samples were immediately placed in icebox which was maintained at temperature of 4 degrees Celsius and transported to either of the two-health centers located in the study area within 12 hours for microscopy examination. Poor specimens were replaced with an immediate spot collection. Registration forms were maintained for documentation of sputum transport details. Data collectors traced suspected individuals with positive interview screening to collect two sputum samples (spot and morning). Furthermore, we cross-checked TB registers at health centers to verify that those suspects who said they were taking anti-TB medication were actually on medication and evaluated thoroughly to ensure that individual new occurrences of TB had not been registered more than once.

The diagnosis of TB was based on the national guideline [10]. Sputum smears were prepared from each spot and morning samples. Smears were air dried, fixed and stained with Ziehl Neelsen (ZN) and examined by direct microscopy for AFB. Positive smears were quantified using International Union against Tuberculosis and Lung Disease (IUATLD) standards [20]. All positive slides and 10% of negative slides were re-confirmed by senior laboratory technician at the University of Gondar teaching hospital laboratory. The overall agreement between the two tests was 97%.

A sputum smear-positive TB case was defined by two sputum smear-positive results for AFB or one positive smear result for AFB with radiographic abnormalities as determined by a clinician or one positive smear results for AFB in the case of HIV positive subjects [10].

The existing National Tuberculosis Control Programme algorithm was used for the diagnosis and treatment of pulmonary TB. Accordingly, all two smear-negative patients clinically investigated and treated by broad spectrum antibiotics for 7–10 day and followed for improvements within 2–4 weeks. Repeated examination of two sputum smears and clinical evaluation were done for individuals with one positive smear and one negative smear. Two sputum smears were repeated for 150 patients who had reported no improvement. All of the patients found negative for both slides and referred to the nearest district hospital for chest X-ray at the discretion of the health officer. Chest X-rays were evaluated by experienced radiologist and 17 patients were diagnosed as smear- negative pulmonary TB. All pulmonary TB cases identified during the survey were treated according to the national guidelines.

Data were double entered using Visual Basic 6.0 (VB 6.0) and Microsoft Access 2003. Analysis was made using Stata/SE 11 for windows (Stata Corp, USA). Odds ratio with 95 percent confidence interval was calculated for some categorical variables in the study to assess the strength of association.

#### Ethical considerations

The study was reviewed and approved by the Institutional Review Board (IRB) of the University of Gondar. Written informed consent was obtained from each participant. Informed written consent regarding eligible subjects below 18 years was obtained from parents or legal guardians. Individual records were coded and accessed only by research staff. Immediate referrals were arranged for those found smear-positive and all were started on anti-TB treatment at nearest health centers in the study area. Others found sick form non-TB causes were referred to the nearest health facility for further investigation and treatment.

## Results

### Study population and TB suspects

A total of 23,590 individuals aged ≥14 years were screened for pulmonary TB through symptom interviews. Of which 11,198 (47.5%) were males and 12,392 (52.5%) were females with male to female sex ratio of 0.9∶1. The mean age (± SD) of respondents was 34 (±17) years, ranging from 14 to 90 years. . About 63% of respondents had no formal schooling. Of the 984 (4.2%) individuals who had a chronic cough for ≥15 days, 831 provided two sputum samples for smear microscopy examination. Sputum samples were not collected and examined in 9.1% (83/914) individuals since they could not provided sputum samples at the time of data collection. Patients detected as smear- positive through active case finding had a mean delay of 13 weeks between having symptom (cough) and diagnosis following our screening. Table 1 shows socio-demographic characteristics of study population.

**Table 1:** Selected socio-demographic characteristics of respondents at Dabat district in Northwest Ethiopia, 2010 (n = 23,590).

Characteristics	Number (%)	P-value
**Sex**		
Male	11190(47.4 )	**0.001**
Female	12400(52.6)	
**Age group( in years)**		
14–29	11384(48.3)	**0.001**
30–44	6078(25.8)	
≥45	6128(25.9)	
**Marital status**		
Single	7855(33.3)	**0.001**
Married	12960 (54.9)	
Divorced/Widowed	2775(11.8)	
**Educational level**		
No formal schooling	14844(62.9)	**0.001**
Primary	4817(20.4)	
Secondary	3208(13.6)	
12+	721(3.1)	
**Residence**		
Urban	5027(21.3)	**0.001**
Rural	18563(78.7)	

### Prevalence of smear-positive TB

**Figure 2:**
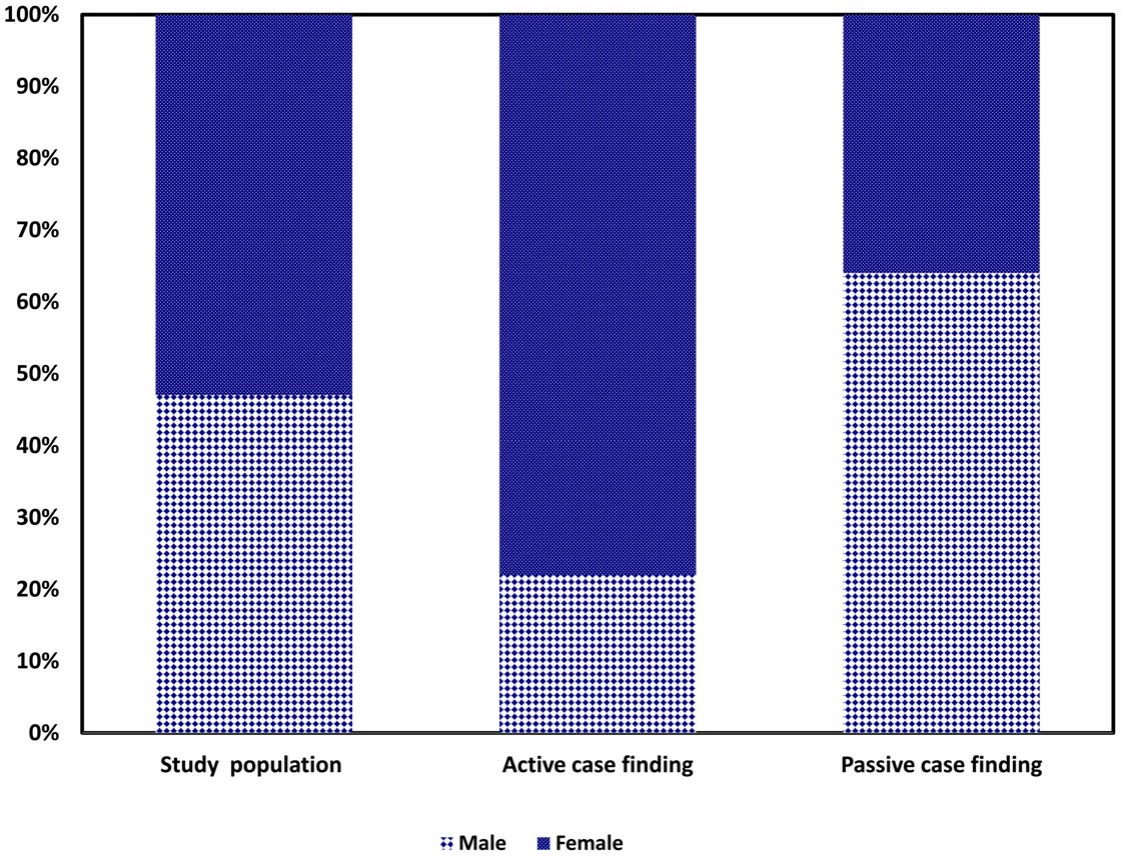
Sex distribution of study population and TB patients identified at Dabat district in Northwest Ethiopia, 2010.

From 984 individuals who reported cough, 150 reported taking anti-TB treatment during the survey. However, a review of TB registers revealed that only 22 smear-positive TB patients were on anti-TB treatment in the same period. Eight hundred and thirty one individuals including the 22 smear-positive TB gave two spot-morning sputum samples for examination. Forty one had new smear positive result, and none of those who were on anti-TB treatment was smear-positive. Thus, the prevalence of new sputum smear-positive TB was 174 per 100, 000 in persons aged ≥14 years (95% CI: 121–227). The ratio of smear-positive cases newly detected by active case finding to those detected through passive case finding was about 2∶1, indicating 2 undiagnosed TB cases in the community for every smear-positive TB case receiving treatment during the survey period (Figure 2). Higher rates of smear-positivity were observed among females [AOR: 3.28, 95% CI (1.54–6.77)], and in the age group ≥45 years [AOR: 2.26, 95% CI (1.12–4.59)] (Table 2).

**Table 2:** Prevalence of smear-positive TB by selected socio-demographic characteristics of respondents at Dabat district in Northwest Ethiopia, 2010 (n = 23,590).

Characteristics	Interviewed	Positive for AFB	COR(95% CI)	AOR(95% CI)
**Sex**				
Male	11190	9	1	1
Female	12400	32	3.21(1.53–6.73)	3.28(1.54–6.77)*
**Age group(years)**				
14–29	11384	14	1	1
30–44	6078	10	1.50(0.77–2.89)	1.29 (0.57–2.91)
≥45	6128	17	1.51(1.05–2.27)	2.26 (1.12–4.59)*
**Marital status**				
Single	7855	8	1	1
Married	12,960	22	1.67(0.74–3.75)	1.35(0.56–3.39)
Divorced/Widowed	2775	11	3.90(1.57–9.72)*	1.85(0.64–5.68)
**Residence**				
Urban	5027	6	1	1
Rural	18563	35	1.58(0.66–3.76)	1.90 (0.96–3.99)

AFB: Acid-fast bacillus; COR: Curd odds ratio; AOR: Adjusted odds ratio; CI: Confidence Interval;

*: Statistically significant, P<0.05.

## Discussion

This population based cross-sectional study identified TB as a significant health problem among individuals aged ≥14 years old. The study revealed that about two-thirds of the symptomatic sputum smear-positive TB cases in the community were undiagnosed and thus untreated. This suggested that for every confirmed pulmonary TB case on treatment there were about two undetected infectious TB cases in the communities.

The prevalence of sputum smear-positive TB in this study is 174/100,000 persons aged ≥14 years. Other prevalence survey reported 78/100,000 in southern Ethiopia [12], 80/100,000 in northwest Ethiopia [13] and 189/100000 in Addis Ababa, Ethiopia [14]. The observed high prevalence of TB in our study in contrast to a very low prevalence documented in rural Ethiopia could be that in our study all sputum smear-negative TB patients were treated for free according to the national guideline [10], which might have increased the participation rate of symptomatic individuals. In addition, the low HIV prevalence in our rural sites, where 79% of our subjects were drawn, might have also contributed to higher frequency of symptomatic presentations with productive cough thus enhancing the chances for making pulmonary TB diagnosis. According to national figures, HIV prevalence is four times higher in urban (7.7%) than in rural (0.9%) areas, while in our study region the prevalence of HIV was six times higher in urban (9.9%) than in rural (1.5%) areas [21].

Wide country variations in the prevalence of smear-positivity have been observed and direct comparison has been recognized to be difficult because of differences in sampling, data collection methods, study population, timing of the study, and related environmental and socioeconomic factors. For example, multistage cluster sampling technique was used in some studies [4], [6], [8]–[9], while others used random sampling [7], [12] or included all samples [5]. The age groups studied were also different among studies and covered all age groups ≥15 years [4], [5], [13], >14 years [12] and ≥14 years [14]. Thus any comparison of prevalence data either within the country or among countries should be done with great caution.

This study observed a significant delay in diagnosis; the mean delay between the onset of pulmonary TB symptoms (mainly cough) and treatment initiation following active detection by this study was 13 weeks. This finding is lower than a mean duration of symptoms of 28 weeks for those identified by active case finding in Bangladesh [5] and higher than study report from Viet Nam (12 weeks) [7]. The reasons for this high patient delay could be lack of awareness on TB and its treatment, reliance on the non-modern healthcare providers, repeated visits to health care facilities without correct diagnosis, cost, distance and transport and cultural interpretations of disease might all surface as important barriers to care in this rural area of Ethiopia [22], [23]. Interventions to improve the quality and accessibility at peripheral health services to facilitate early TB diagnosis and treatment remain very important.

Routine reports based on passive case finding from health care facilities have repeatedly shown higher prevalence of TB for men than women [1], [5], [11]. However, in our study the prevalence of smear-positive TB cases detected through active case finding was 3.3 times higher for females than males. This finding is consistent with data from rural areas of Vietnam [7] and Southern Ethiopia [24]. This could be due to the fact that, active case finding via household visits reduces the social inequity in accessing health services. Interventions to address gender inequalities are essential to increase TB case detection [5], [13],[25],[26].

Study from Guinea-Bissau and three West Africa countries reported that increase in age was significantly associated with TB infection (P<0.0001) [27], [28]. Our study revealed that the prevalence of smear- positive TB was 2.3 times higher in older persons. It is known that the risks of TB acquisition, progression and reactivation increase as age increases [29]. Additionally, older persons may not visit health services because of long walking distance. In this survey we included individuals aged ≥14 years. Smear-positivity is infrequent in those <14 years of age and collection of sputum samples is difficult; exclusion of this group will reduce workload but have only a small effect on the prevalence estimate for the total population [16], [17].

Smear-positive TB case detection rate is 36% in Ethiopia [11]. Lack of accurate and rapid diagnostics remains a major obstacle to progress in this regard. Health care facilities still heavily rely on sputum smear microscopy for the diagnosis of TB. This technique has low sensitivity and specificity [30], however, efforts were made to ensure quality AFB diagnosis through appropriate instruction of symptomatic individuals on how to produce quality sputum sample from their lung. In the laboratory the macroscopic appearance of a sputum sample was checked and poor specimens were replaced with an immediate spot collection.

The accurate denominator provided by the Dabat HDSS for this study is a unique strength for this study. Furthermore, Geographic Information System (GIS) based mapping is ongoing within Dabat HDSS to link disease patterns to geographic profiles which can generate hypotheses around host spots for TB transmission (such as hotspots for TB), and provide opportunities for targeted interventions such as intensified case-finding [31], [32], [33]. This can be done through the innovative Health Extension Program (HEP) of the country that focuses on the prevention and control of communicable diseases in including TB diagnosis and treatment [34]. Further, our estimate is robust as it used well trained data collectors using a simple and validated questionnaire contained selected high sensitive questions to pick suspects of pulmonary TB cases.

One of the limitations to our study is not doing sputum culture. Ideally, in TB prevalence survey sputum smear and culture should be performed on all screened individuals [35]. The prevalence of TB may be underestimated by 37% if only symptoms are used to identify TB suspects. Therefore, the strategy we applied cannot detect smear negative but culture-positive TB cases. The TB prevalence studies used sputum screening and sputum culture for confirmation, the chances of detecting smear positive TB cases were significantly greater [7]. However TB prevalence surveys in resource constrained countries need to take logistical and financial limitations to offset the criticism of spending the meager resources on research. Although our screening strategy has limitations with the possibility of underestimation, suggesting there are probably more TB cases in the surveyed communities than reported in this study. Furthermore, sputum samples were not collected and examined in 9.1% of the symptomatic individuals that may further aggravate the underestimation of the true TB prevalence in our study. With a better screening method and diagnostic facility, this prevalence could even be more than what is reported. However, one thing is very certain; the passive case detection approach currently implemented in Ethiopia leaves many undetected and thus untreated TB cases in the community perhaps favoring the continuous spread of the disease.

In conclusion, about two-thirds of smear-positive TB cases remain undiagnosed and untreated creating a cluster of infectious cases in the community that maintain active TB transmission. Thus, the low case detection rate observed nationally can be improved by introducing enhanced case detection mechanisms and promoting favorable health seeking behaviors especially among females and older age groups at the community level using community based health infrastructure.
